# Cooperation between two modes for DNA replication initiation in the archaeon *Thermococcus barophilus*

**DOI:** 10.1128/mbio.03200-23

**Published:** 2024-02-29

**Authors:** Logan Mc Teer, Yann Moalic, Valérie Cueff-Gauchard, Ryan Catchpole, Gaëlle Hogrel, Yang Lu, Sébastien Laurent, Marie Hemon, Johanne Aubé, Elodie Leroy, Erwan Roussel, Jacques Oberto, Didier Flament, Rémi Dulermo

**Affiliations:** 1 Univ Brest, Ifremer, CNRS, UMR6197 Biologie et Ecologie des Ecosystèmes marins Profonds (BEEP), Plouzané, France; 2 LabISEN, Yncréa Ouest, Brest, France; 3 Université Paris-Saclay, CEA, CNRS, Institute for Integrative Biology of the Cell (I2BC), Gif-sur-Yvette, France; Institut Pasteur, Paris, France

**Keywords:** DNA replication, homologous recombination, DNA-seq, recombination-dependent replication (RDR), log/stationary phases, recA/Rad51 protein family, *Ori*, Cdc6

## Abstract

The mechanisms underpinning the replication of genomic DNA have recently been challenged in *Archaea*. Indeed, the lack of origin of replication has no deleterious effect on growth, suggesting that replication initiation relies on homologous recombination. Recombination-dependent replication (RDR) appears to be based on the recombinase RadA, which is of absolute requirement when no initiation origins are detected. The origin of this flexibility in the initiation of replication and the extent to which it is used in nature are yet to be understood. Here, we followed the process of DNA replication throughout the growth stages of *Thermococcus barophilus*. We combined deep sequencing and genetics to elucidate the dynamics of *oriC* utilization according to growth phases. We discovered that in *T. barophilus*, the use of *oriC* diminishes from the lag to the middle of the log phase, and subsequently increases gradually upon entering the stationary phase. Although *oriC* demonstrates no indispensability, RadA does exhibit essentiality. Notably, a knockdown mutant strain provides confirmation of the pivotal role of RadA in RDR for the first time. Thus, we demonstrate the existence of a tight combination between *oriC* utilization and homologous recombination to initiate DNA replication along the growth phases. Overall, this study demonstrates how diverse physiological states can influence the initiation of DNA replication, offering insights into how environmental sensing might impact this fundamental mechanism of life.

**IMPORTANCE:**

Replication of DNA is highly important in all organisms. It initiates at a specific locus called *ori*, which serves as the binding site for scaffold proteins—either Cdc6 or DnaA—depending on the domain of life. However, recent studies have shown that the *Archaea*, *Haloferax volcanii* and *Thermococcus kodakarensis* could subsist without *ori*. Recombination-dependent replication (RDR), via the recombinase RadA, is the mechanism that uses homologous recombination to initiate DNA replication. The extent to which *ori*’s use is necessary in natural growth remains to be characterized. In this study, using *Thermococcus barophilus*, we demonstrated that DNA replication initiation relies on both *oriC* and RDR throughout its physiological growth, each to varying degrees depending on the phase. Notably, a knockdown RadA mutant confirmed the prominent use of RDR during the log phase. Moreover, the study of ploidy in *oriC* and *radA* mutant strains showed that the number of chromosomes per cell is a critical proxy for ensuring proper growth and cell survival.

## INTRODUCTION

DNA replication is an essential process for all cells, allowing DNA duplication before cell division. This process begins with the recognition of a specific DNA sequence (*oriC*) by an initiator protein, which promotes the opening of the DNA double helix. Archaeal chromosomal DNA replication is largely homologous to that of eukaryotes and differs notably from its bacterial counterpart (1, 2). In most *Archaea*, replication origins consist of a cluster of ORB sequences, which are bound by the initiator protein Orc1/Cdc6 to initiate replication (3–5).

Unlike bacterial chromosomes, which typically harbor a single *oriC* sequence (6), archaeal genomes harbor a variable and species-specific number of origins. *Pyrococcus abyssi* and *Nitrosopumilus maritimus* display a single chromosomal replication origin; whereas, *Sulfolobus acidocaldarius* and *Saccharolobus solfataricus* (previously *Sulfolobus solfataricus*) carry three and *Pyrobaculum calidifontis*, four (7–11). Recently, the non-essential nature of *oriC* has been observed for some euryarchaeal polyploid species (12–14), even if some *Archaea* such as *Haloferax mediterraneii* still require at least one *oriC* to be viable (15). The four origins of *Haloferax volcanii* (DS2 strain) and the single origin of *Thermococcus kodakarensis* can be deleted (13, 14). A slight increase in growth rate was observed for the multiple *ori*-depleted strain of *H. volcanii*, questioning the role and maintenance of these origins (14). Although *ori*-depleted *T. kodakarensis* growth rates were unaffected, it displayed a decrease in long-term viability (13). Concerning the initiator protein, the *ori*-binding protein Orc1/Cdc6 can be removed in *T. kodakarensis* (13), similar to *DnaA* in cyanobacteria (16). However, it is not possible to delete all *orc1/cdc6*-encoding genes from *H. volcanii* (H26 strain [17]). Several mechanisms have been proposed for the Cdc6/DnaA-independent initiation of replication. These include rolling circle replication of plasmids by Rep proteins (18), iSDR (inducible stable DNA replication), and cSDR (constitutive stable DNA replication) (19, 20). iSDR is a particular form of recombination-dependent replication (RDR) induced in *Escherichia coli* during the SOS response, initiating chromosomal replication from D-loops (intermediates of homologous recombination). In contrast, cSDR occurs in *E. coli* RNaseH mutants, where RNA transcripts invade the DNA duplex, creating an R-loop that initiates replication. Both iSDR and cSDR require homologous recombination proteins such as RecA and PriA to ensure DNA replication (19, 20). Consistently, it was proposed that RDR could operate in *H. volcanii* since *RadA* became essential in the strain deleted of all four *oriC* (14). RDR was first described for T4 phage replication and functions via loop formation after strand invasion to initiate DNA replication. T4 homologous recombination proteins are essential to perform this function (21).

The ability of some archaeal species to survive without *oriC* raises numerous questions, such as the stable maintenance of non-essential origins, the mechanism by which replication occurs in the absence of functional origins, and the disparity in the essential/dispensable nature of origins between species. Clearly, DNA replication initiation in the Archaeal domain remains mysterious in several aspects. To investigate the role of *oriC* and RadA in different features of the archaeal life cycle, we used the anaerobic and non-obligate piezophilic Archaeon *Thermococcus barophilus* MP. This Euryarchaeal species was isolated from deep-sea hydrothermal vents (22) and is genetically tractable (23, 24). We demonstrate the flexible utilization of *oriC* all along the growth stages, with a reduced use at the beginning of the log phase. This versatility could be directly linked to RDR after conducting *radA* knockdown experiments. This work demonstrates for the first time the adaptation of DNA replication initiation to the physiological state of a cell.

## RESULTS

### *oriC* activation correlates with cell growth rate in Thermococcales

**Fig 1:**
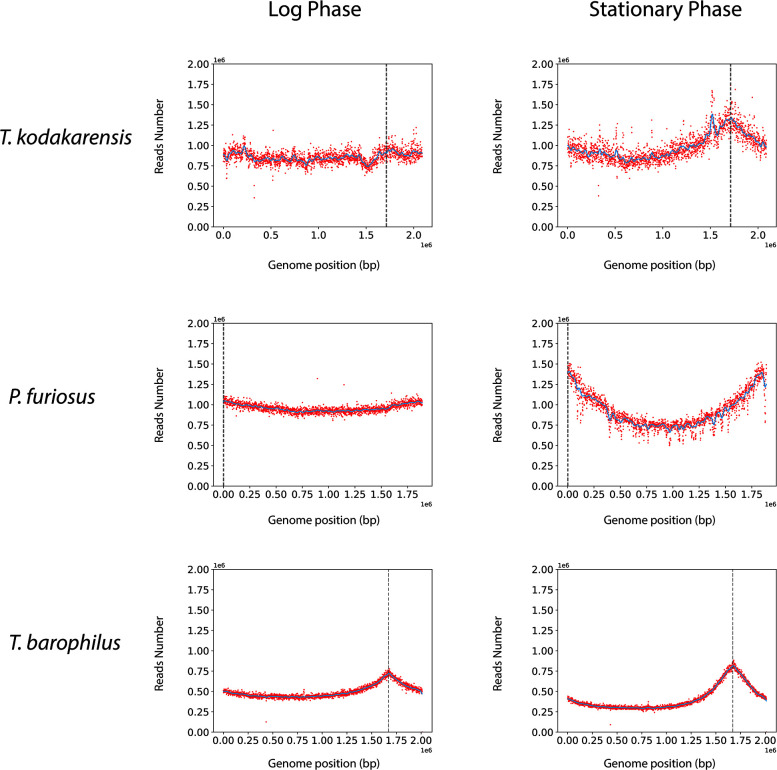
Marker frequency analysis of *T. kodakarensis*, *P. furiosus*, and *T. barophilus* genomes during exponential and stationary phases. Blue lines represent the one-dimensional Gaussian filter. Vertical dotted lines represent canonical *oriC* localization on genomes.

Given a recent work indicating that the chromosomal origin of replication is not used in the archaeon model *T. kodakarensis*, we started by assessing whether this feature is shared by other *Thermococcales*. Thus, three model species were chosen to investigate *oriC* detection through deep sequencing at log and stationary phases: *T. kodakarensis* KOD1, *Pyrococcus furiosus* DSM3638, and *T. barophilus* MP (here, its genetic strain Δ*TERMP_00517* will be referred as wild type [WT]) (Fig. 1). Interestingly, marker frequency analysis (MFA) curves showed peaks at stationary phase for each of the three species (respectively at positions: 1,712,000, 0, and 1,671,000 bp). The peaks indicated the precise position of the canonical *oriC* defined in *Thermococcales* as an intergenic region, containing ORB sequences and close to *radA* and *cdc6* genes (5, 25, 26). At the log phase, no peak was detected for *T. kodakarensis* while a weak peak was still present for *P. furiosus* and *T. barophilus*; whereas a higher peak was observed for the three Thermococcales during the stationary phase. These results highlighted a differential use of *oriC* during growth in these three Thermococcales models. Because a different profile was detected in *T. kodakarensis* (no peak at *oriC* during stationary phase) (13), we suspected that the timing of DNA extraction strongly influenced the MFA profile, meaning that the physiological state was decisive for the use of *oriC* during DNA replication initiation. Subsequently, we subjected our model, *T. barophilus*, to genetic modifications involving *oriC* and RadA, monitoring MFA at eight different points across a kinetic curve (details to be discussed later).

### *oriC* is not essential for *T. barophilus*

**Fig 2:**
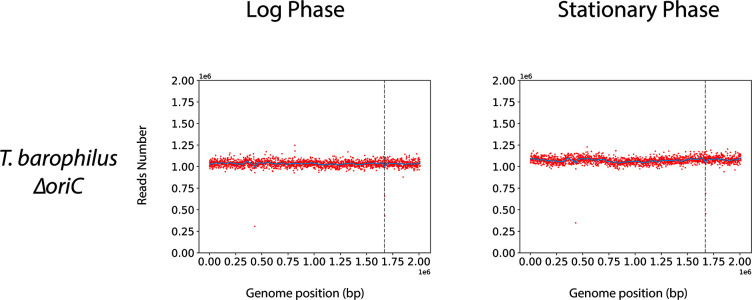
Marker frequency analysis of *oriC* mutant. Blue lines represent the one-dimensional Gaussian filter. Vertical dotted lines represent canonical *oriC* localization on genomes.

It was previously shown that the origin of replication is not essential for viability in some *Archaea*, e.g., *H. volcanii* (14). Similarly, a recent work reported that the chromosomal replication origin (*oriC*) is non-essential in the archaeon *T. kodakarensis* (13). On the contrary, *H. mediterraneii* requires at least one origin of replication to be viable (15). Here, *oriC* could be deleted and MFA confirmed that this mutant strain no longer uses a detectable *oriC* (Fig. 2), indicating that *oriC* was the sole active *oriC* in *T. barophilus*. Similar results were obtained with ∆*Tbcdc6* (data not shown). The viability of *T. barophilus* in the absence of *Tbcdc6* or *oriC* was similar to that observed for *T. kodakarensis* (13) and *H. volcanii* (14), thereby confirming the existence of an alternative pathway to initiate DNA replication. Moreover, the decrease in the height of the peak in the exponential phase for WT cells could be due to the involvement of this alternative pathway at this stage.

### Reduction in RadA expression in *T. barophilus*

**Fig 3:**
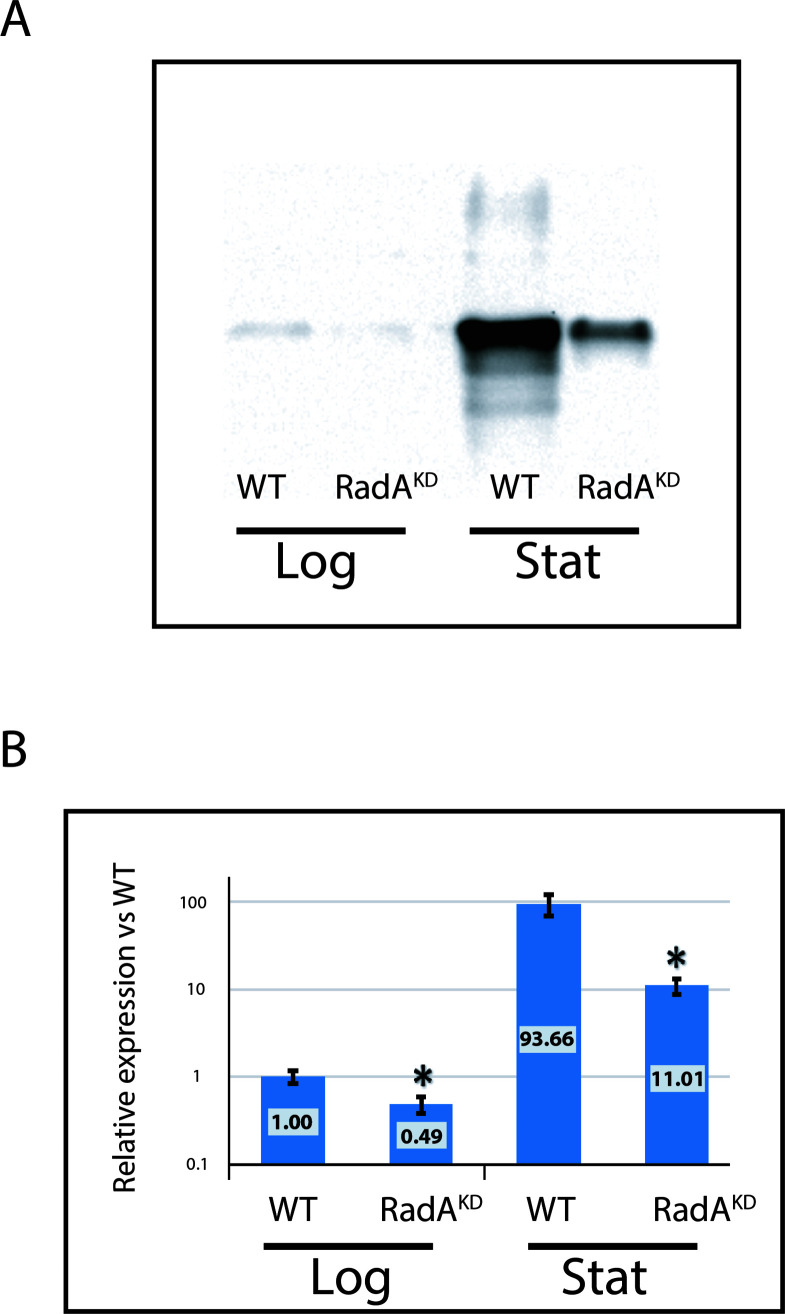
Expression of RadA strongly decreases in RadA^KD^ strain. (**A**) Western blot on RadA in WT and RadA^KD^ strains during exponential and stationary phases. A representative of the result of three different experiments is shown here. Each line was performed using 5 µg of total proteins. (**B**) Quantification of the western blot on three different experiments. Normalization was done on the WT at exponential phase.

To confirm whether recombination serves as an alternative pathway for DNA replication initiation in lieu of *ori*, we attempted to delete *radA*. However, similar to other hyperthermophilic *Archaea* such as *T. kodakarensis* and *P. furiosus* (13, 27, 28), we failed to eliminate this gene in *T. barophilus*, suggesting its essential nature. To overcome this, we attempted to decrease the expression of the *radA* gene by exchanging its promoter with that of a weakly expressed gene. For that purpose, we used RNA seq analysis data and identified the promoter of gene *TERMP_00015* (annotated as L-threonine 3-dehydrogenase) that showed, depending on the growth phase, between 39- and 64-fold lower expression than *radA* (Data set GSE229955). Following the creation of the knockdown mutant (named RadA^KD^), we measured its expression through western blotting (refer to Fig. 3A and B), verifying a significant reduction in the RadA protein in this mutant. Interestingly, western blot analyses showed that RadA is weakly expressed during the exponential phase; whereas, it is 94 times more expressed during the stationary phase in WT. In the knockdown mutant, RadA was twofold (*P* < 0.034) to 8.5-fold (*P* < 0.023) less expressed compared to the WT strain (Fig. 3B).

### Reducing RadA expression increases *oriC* utilization

**Fig 4:**
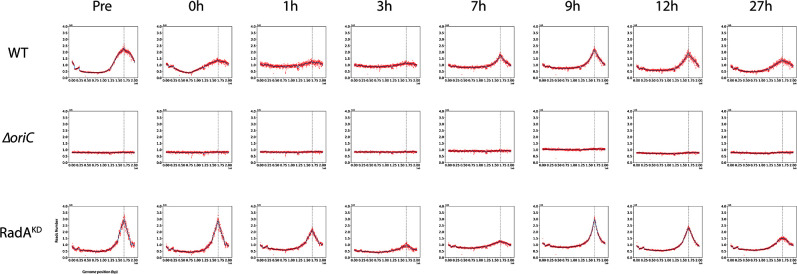
Marker frequency analysis at different times of the growth of ∆*oriC*, RadA^KD^, and WT. Blue lines represent the one-dimensional Gaussian filter. Vertical dotted lines represent canonical *oriC* localization on genomes. Pre means preculture/inoculum. Here, only one curve per points is shown; all data are given in Fig. S1, S2, and S3.

**Fig 5:**
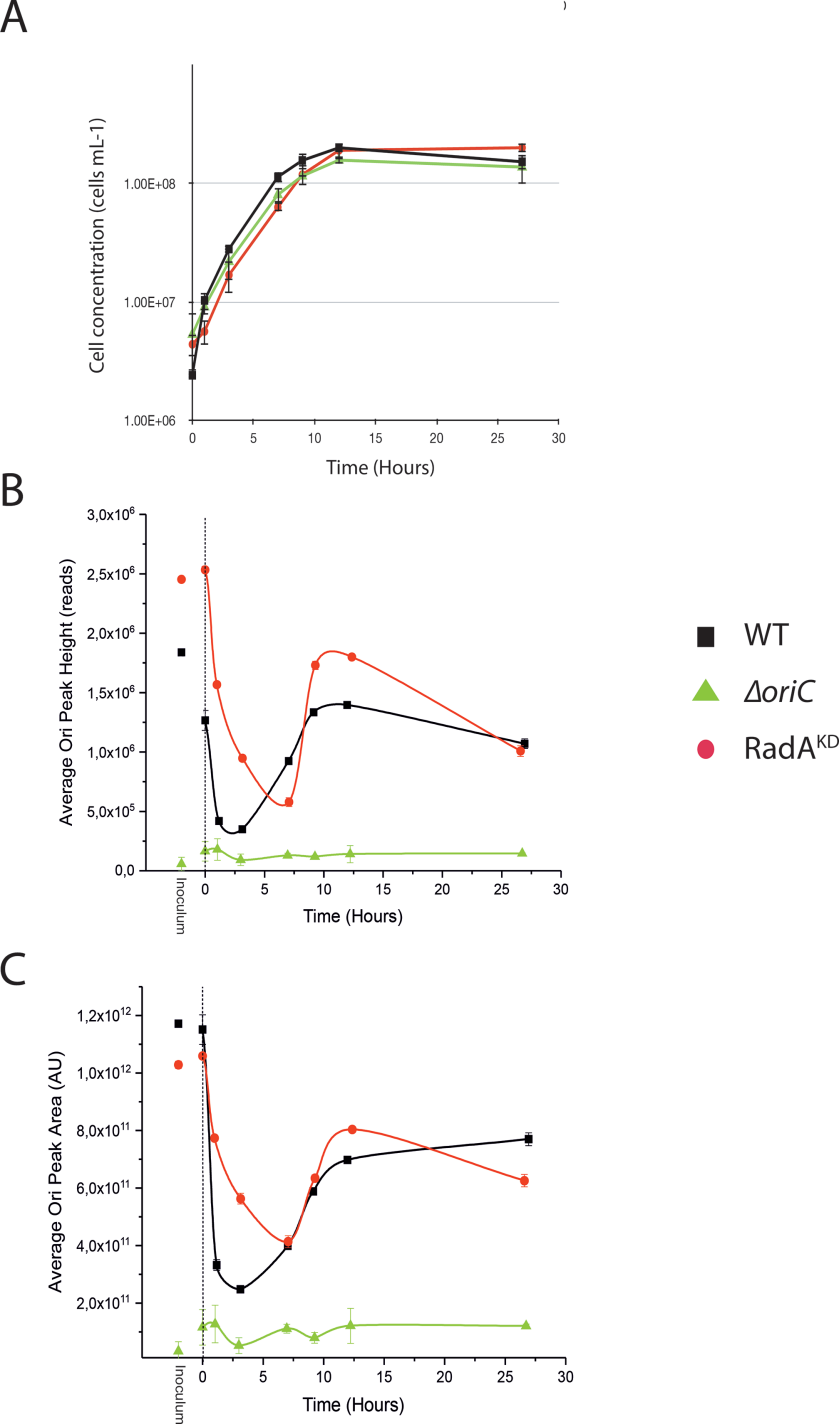
Growth and analysis of the characteristic of the MFA peaks at different times of the growth of ∆*oriC*, RadA^KD^, and WT. (**A**) Growth curve of WT, Δ*oriC*, and RadA^KD^ strains at 85°C and 0.1 MPa. The data presented here are the average of three independent experiments with error bars representing standard deviation. (**B**) Analyses of the height of the MFA peaks observed at each sample point, based on Fig. S1, S2, and S3. (**C**) Analyses of the area of the MFA peaks observed at each sample point, based on Fig. S1, S2, and S3.

The availability of these two mutant strains allowed us to examine more closely the use of *oriC* throughout the distinct growth phases in batch cultures. MFA was performed at different key moments of the growth of these strains (Fig. 4, detailed in Fig. S1, S2, and S3). The MFA profiles were analyzed by computation of peak height and area at the *oriC* location (Fig. 5). As a foreword to this analysis, the growth curves of the three strains were similar (at 85°C, 0.1 MPa) (Fig. 5A). Beginning with the WT strain, the use of *oriC* diminished during the initial 0–3 h of growth kinetics, and subsequently, it gradually increased as it approached the stationary phase (from 7 to 12 h), confirming the earlier findings illustrated in Figure 1 (Fig. 4; Fig. S1). This becomes more evident when examining the black curves representing the *oriC* peak height and area (Fig. 5B and C). Conversely, as anticipated, the *oriC* mutant exhibited no *oriC* utilization (Fig. 4, 5B and C; Fig. S2). On the other hand, the *radA* mutant demonstrated a profile more similar to that of the WT, albeit with *oriC* utilization that was consistently higher based on both peak height and area (Fig. 5B and C). This indicates an increased reliance on *oriC* for replication initiation, a perspective that aligns with a diminished direct involvement of RDR, a correlation attributed to the decreased expression of RadA in RadA^KD^.

### Cell growth, replication mode, and ploidy

**Fig 6:**
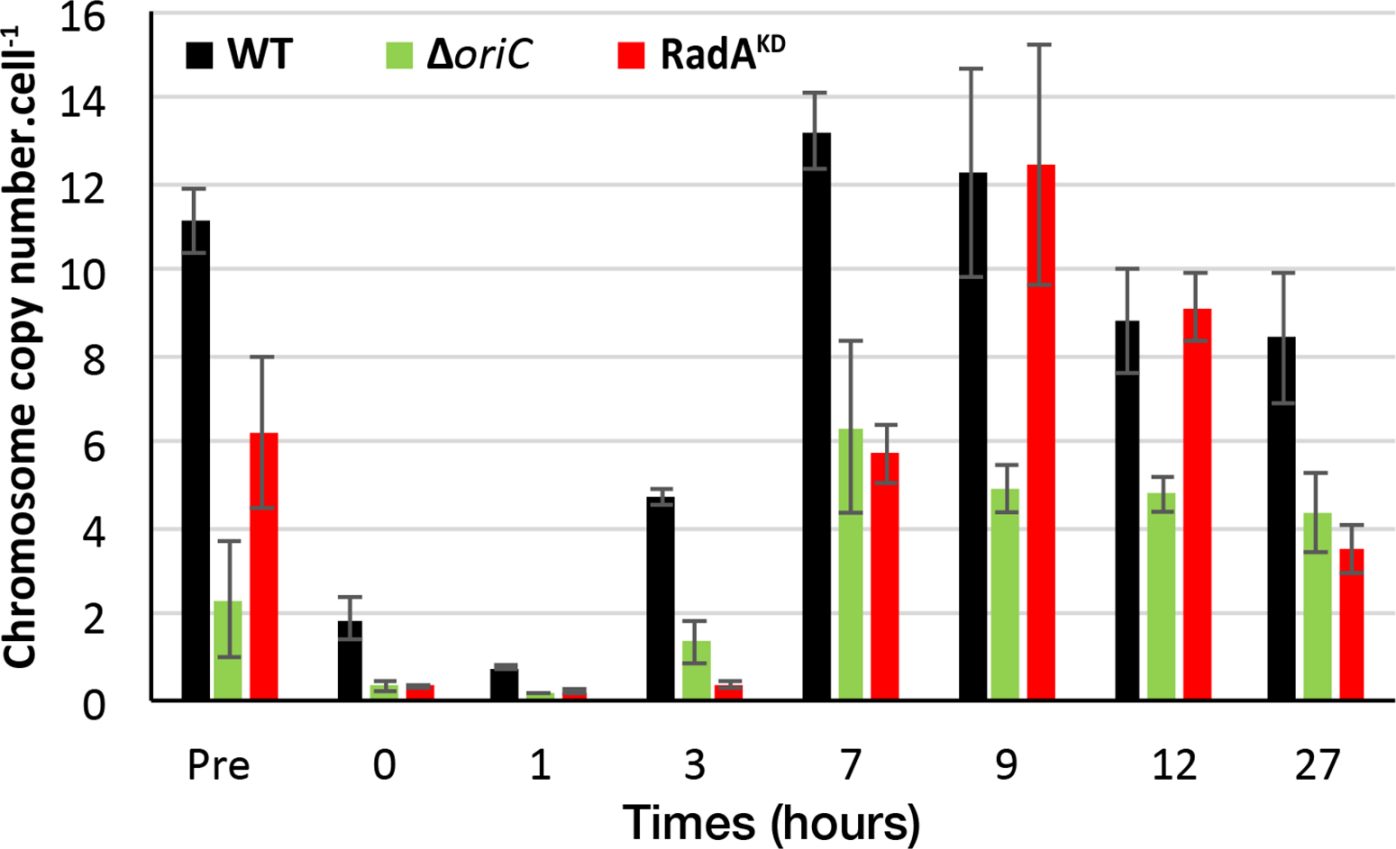
Ploidy observed at different times of the growth of ∆*oriC*, RadA^KD^, and WT. The data presented here are the average of three independent experiments obtained by qPCR with error bars representing standard deviation.

Given the proposition that monoploïd cells need an origin-based mechanism of DNA replication to ensure their survival during an extended stationary phase (13), we have supplemented our experiments with qPCR, enabling the quantification of chromosome numbers during the growth period (Fig. 6). Similarly to a previous publication (29), we found that WT *T. barophilus* contained up to 13 chromosomal copies at the end of log phase, decreasing to 8 copies at the stationary phase (Fig. 6). During the initial stages of growth (0, 1, and 3 h), caution is advised when interpreting the low ploidy levels detected, which may occasionally fall below one copy. This is attributed to the potential loss of a significant number of cells and, consequently, DNA during these early time points. This was probably due to the high proportion of colloidal sulfur present in the medium that renders difficult the cells harvesting at the beginning of culture. The peak ploidy for all three strains occurred at 7 h (for *oriC* mutant and WT) or 9 h (for RadA^KD^) of growth. However, the maximal ploidy for ∆*oriC* was limited to approximately 6 copies, which was half as much as compared the other two strains. In addition, a high quantity of RadA appeared to be required to conserve a relatively high ploidy during stationary phase. Altogether, these results indicated a dual reliance on *oriC* and RDR in replication, enabling an efficient chromosome production during the log phase and sustained maintenance of a high chromosome copy number during the stationary phase. Here, the absence of *oriC* prevented attaining a copy number equivalent to that of the WT. Taken together, these results demonstrate that the mode of replication initiation induces fluctuation in ploidy across different growth phases.

### Effect of high hydrostatic pressure (HHP) on replication initiation

**Fig 7:**
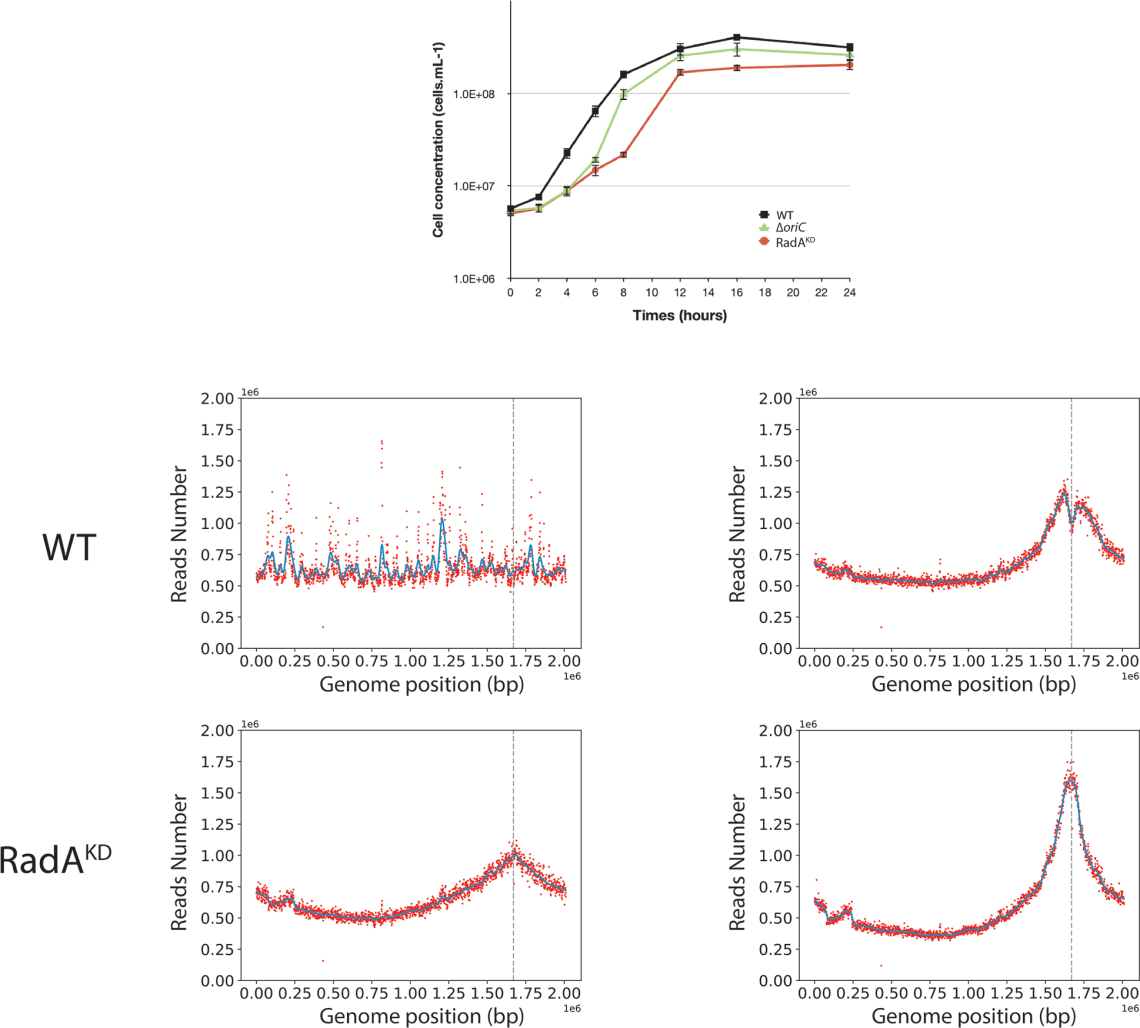
Growth of ∆*oriC*, RadA^KD^, and WT at 85°C and 40 MPa, and MFA associated at exponential and stationary phases. (**A**) Growth curve of WT, Δ*oriC*, and RadA^KD^ strains at 85°C and 40 MPa. (**B**) Marker frequency analysis of WT and RadA^KD^ genomes during exponential and stationary phases. Blue lines represent the one-dimensional Gaussian filter. Vertical dotted lines represent canonical *oriC* localization on genomes.

HHP is a notable physical parameter present in the natural deep-sea hydrothermal vent ecosystem of *T. barophilus*, which is classified as a piezophile (22, 30). As *T. barophilus* growth is improved at 40 MPa, its optimal pressure, all following experiments were performed under this condition with the same genetic strains (Fig. 7A). First, it is apparent that RadA^KD^ growth was negatively affected by pressure as the doubling time was approximately 2.4 times higher than the reference strain under these conditions (Fig. 7A; 90.6 ± 7.5 min vs 215.3 ± 35.7 min), whereas Δ*oriC* occupies an intermediate position (Fig. 7A). To verify that this phenotype could be linked to RadA expression levels, western blot experiments were carried out on cell extracts originating from log and stationary phases at 40 MPa (Fig. S4). At this optimal pressure condition, the quantity of RadA follows the same pattern than at atmospheric pressure, namely, a higher level at stationary than at log phase (Fig. S4, lane 6 vs lane 5). This is also true for the RadA^KD^ strain (lane 8 vs lane 7). However, it is noteworthy that the WT strain seemed to require less RadA, especially during the stationary phase (lane 6 vs lane 2). We then performed MFA on the strains except ∆*oriC* (Fig. 7B). Although these strains did not display distinct MFA profiles during the stationary phase (both demonstrating robust *oriC* utilization), only the RadA^KD^ strain exhibited *oriC* use during the log phase (Fig. 7B). This result indicates that (i) *oriC* is less used under optimal growth conditions than at atmospheric pressure (0.1 MPa), and (ii) the absence of RadA significantly enhances *oriC* utilization under pressure. Moreover, it is noteworthy that in the stationary MFA of the WT strain, there is a curve inversion at the *oriC* coordinate, forming a convex shape with its vertex precisely located at this point. This particular shape was observed in three different experiments exclusively for the WT strain at 40 MPa. We checked that this particular shape does not account for a genomic inversion; however, we believe that this reduction of reads detection centered at *oriC* locus does not contradict its use as the initiation point because of its symmetrical shape. We hypothesized that a certain part of the reads is absent due to some issues in DNA/chromatin availability at this area during DNA-seq process in this particular condition.

## DISCUSSION

In this study, we analyzed the initiation of DNA replication in *T. barophilus* all along the growth phases. Our results highlight the cooperation between two initiation modes. We observed that *oriC* initiation is primarily active from the late log to the stationary phases. On the contrary, we noted a reduction in *oriC* activation from early to mid-log phases (MFA, Fig. 1; Fig. S1), which is related to RadA availability (Fig. 3 and 4; Fig. S3). This greatly strengthens the hypothesis that RDR is an alternative pathway for replication initiation in *Archaea* (13, 14). It is interesting to note that the reduction of RadA has varied impacts on the growth rate depending on the pressure conditions (Fig. 5 and 7). We hypothesize that the absence of growth impact at non-optimal pressure (0.1 MPa) could be associated with the growth rate limitation encountered under these conditions and does not require a combination of two replication initiation modes to reach its maximum. At 40 MPa, the growth rate is higher and appears to require the implication of two modes of initiation to ensure a sufficient level of replication. Moreover, when comparing RadA^KD^ to ∆*oriC*, RDR seems to be more important than *oriC* to reach such a high rate at 40 MPa. To sum up, a faster cellular division cannot be achieved with only one origin of replication. It would be interesting to evaluate ploidy under optimal conditions to observe its behavior at 40 MPa. However, at 0.1 MPa, the difference in ploidy did not exhibit any growth distinctions between the three strains (Fig. 5). Finally, our results questioned the meaning of “optimal” conditions that are tightly linked to the growth rate and, thus, the capacity of the cell to improve DNA replication initiation. Here, we demonstrated that it could be linked to the capacity of the cell to accelerate its DNA replication by using several modes of initiation. Examining the MFA profiles, it appears that RDR initiates replication at sufficient number of sites, rendering them undetectable by this visualization technique (Fig. 4; Fig. S2). Consequently, the rationale behind retaining an *oriC* that accounts for only one site to initiate replication should extend beyond a simple replication mechanism. Gehring et al. (13) found that a decrease in the viability of Δ*oriC* Δ*cdc6 T. kodakarensis* strain was observed when the stationary phase was prolonged. This observation could be linked to a decrease in ploidy within the *T. kodakarensis* mutant, similar to what we observed in the *oriC* mutant in *T. barophilus*. These results demonstrate that *oriC* plays a key role in ploidy maintenance to ensure cell survival during longer stationary phases which are the main physiological states in their natural habitat (31). Moreover, our result could explain why RadA is much more abundant in the stationary phase compared with the exponential phase in the WT strain (Fig. 3). Indeed, RadA seems to stabilize the ploidy during the late stationary phase (Fig. 6, 27 h). A nine-time reduction in its quantity has an important impact on the number of chromosomes per cell even in the presence of *ori*. In addition, all the attempts to build a strain depleted of *cdc6* combined with a RadA^KD^ have failed, reinforcing the crucial role of RadA to initiate the replication and indicating that there is no other pathway to initiate DNA replication.

It seems unlikely that *Archaea* replicate via an iSDR mechanism, as iSDR-dependent cells are unable to form colonies in *E. coli* (20). Moreover, cSDR, which used R-Loop, might not be used in *Archaea*, because RNaseH is intact in the studied *cdc6* or *oriC* mutants of *Archaea* (including the published *T. kodakarensis* and *H. volcanii*, and our *T. barophilus* strains), suggesting that an alternative form of DNA replication initiation may exist in *Archaea*. It is worth mentioning that Δ*oriC* strains of *T. kodakarensis* and *H. volcanii* require the homologous recombination protein RadA for survival. In our study, we observed that a RadA^KD^ strain uses more its *oriC* in different growth culture conditions, strongly suggesting that the lack of RadA forces the cell to rely more on *oriC* for initiating DNA replication. Our findings, in conjunction with the previously mentioned studies, demonstrate for the first time in *Archaea* that RadA actively contributes to the initiation of DNA replication, strongly indicating the existence of RDR in *Archaea*. Given the distinct behaviors of different *Archaea* regarding *oriC* utilization, this implies that the regulation of *ori*/RDR utilization varies between strains. Some, like *H. volcanii*, primarily use *ori*; whereas others, such as *T. kodakarensis*, predominantly use RDR. Strains such as *T. barophilus* exhibit a combination of both mechanisms to initiate DNA replication. In fact, the peak observed at stationary phase for *T. kodakarensis* in our study is probably consecutive to our experimental conditions, which differ from those used by Gehring et al. (13), highlighting the impact of environment on replication. It could also be interesting to examine the phenotype of WT and *oriC* mutant in *H. volcanii* and *T. kodakarensis* under different conditions to see if as *T. barophilus*, deviating from the environmental optima, shows any differences in *oriC* utilization. In the “Replicon Theory,” Jacob and Brenner hypothesized that the initiation of DNA replication requires a replicon consisting of a replicator sequence, the origin, and a gene encoding an initiator protein (32). This theory was proven to be correct for most living organisms, although alternative forms of replication would operate in some particular cases as we observed for some *Archaea*.

Although our study contributes to addressing why seemingly dispensable *oriC* are retained in Thermococcales, it also raises additional questions, such as how ploidy is regulated in *Archaea* and the mechanisms governing *ori*-independent replication. In this case, the way in which the external cellular environment is able to influence the replication mechanism has yet to be characterized. Future experiments will focus on the identification of the molecular pathways involved in these mechanisms as well as their regulation. Notably, experiments able to follow the replisome progression (33) as well as RadA localization along the chromosome will help to get a better understanding of replication initiation timing. A special emphasis on RadA functions during the growth phases should be achieved in order to explain its quantitative difference between the stationary and the log phases. It would be also interesting to see if the decrease of ploidy observed in ∆*oriC* strain induces a gene dosage regulation leading to some global gene expression modifications that could lead to a selective disadvantage of low ploidy.

## MATERIALS AND METHODS

### Strains, media, and growth conditions

Bacterial and archaeal strains are listed in Table 1 . *E. coli* strain DH5α was the general cloning host. Luria-Bertani (LB) broth was used to cultivate *E. coli. Thermococcales*-rich medium (TRM) was used to cultivate Thermococcales, under anaerobic condition and at 85°C as described in Zeng et al. (34). Media were supplemented with the appropriate antibiotics used at the following concentrations: ampicillin 25 µg mL^−1^ for *E. coli*, simvastatin 2.5 µg mL^−1^, and 6MP (100 µM) for *T. barophilus*. Then, necessary elemental or colloidal sulfur (0.1% or 0.5 g/L final concentration) was added for *Thermococcales*. Plating was performed by addition to the liquid medium of 16 g L^−1^ of agar for *E. coli* and 10 g L^−1^ of phytagel for *T. barophilus*.

**TABLE 1:** Strains and plasmids

Strain or plasmid	Genotype or other relevant characteristics	Source or reference
Strains		
*E. coli*		
DH5α	Φ*80dlacZ*Δ*m15, recA1, endA1, gyrA96, thi-1, hsdR17 (r_k_−, m_k_+), supE44, relA1, deoR,* Δ(*lacZYA-argF)U169*	Thermo Fisher Scientific, Asnières, France
*T. barophilus*		
UBOCC-M-3300	Δ*TERMP_00517*	(23)
RDMP44	Δ*TERMP_00517* Δ*oriC*	This study
RDMP45	Δ*TERMP_00517* Δ*cdc6*	This study
RDMP74	Δ*TERMP_00517* p*TERMP_00015*::*radA*	This study
*T. kodakarensis*		
KOD1	WT	UBOCC-M-3203
*P. furiosus*		
DSM3638	WT	UBOCC-M-2923
Plasmids		
pUPH	Pop-in pop-out vector	(23)
pRD236	pUPH + *oriC* UpDn	This study
pRD265	pUPH-*cdc6c* UpDn	This study
pRD423	pUPH-p*TERMP_00015*::*radA* UpDn	This study

### Plasmid construction

Primers are given in Table 2 . Deletion of *oriC* and *cdc6* was performed using pRD236 and pRD265. These plasmids were constructed using primer pairs 145/250, 148/249 and 298/299, and 300/301, respectively. Fragments generated by PCR were fused using primer pairs 145/148 and 298/301, respectively. Then, these fusions were inserted into pUPH using *Kpn*I and *Bam*HI restriction sites.

**TABLE 2:** Primers

Primer name	Sequence	Utilization
145	GCTAGGATCCGGGGTGAATCAATGAGCCTTGC	To delete *oriC*
148	TCAGGGTACCATTTCCCTGACCCTCCAGTGG	
249	CAAAAGAAGTAAAGTTGATTTTGGACGAATGAATTCCTAAAATTATATTTTAAAGGACAAATGCTAATATTTCTCTGG	
250	CCAGAGAAATATTAGCATTTGTCCTTTAAAATATAATTTTAGGAATTCATTCGTCCAAAATCAACTTTACTTCTTTTG	
257	GGCTGCCTCTCCTTCGGG	To analyze the deletion of *oriC*
258	GCAATTCTTTTGGAGTATAGCTATGTCTAAGG	
298	GCTAGGATCCAACAAGTCATTCAGTGGCTGAGGG	To delete *cdc6*
299	CGAGCTCATTTATTAGATCACTGACCCTTCTTCCCTGACCCTCCAGTGGAAACATAGCC	
300	GGCTATGTTTCCACTGGAGGGTCAGGGAAGAAGGGTCAGTGATCTAATAAATGAGCTCG	
301	TCAGGGTACCTAGTTCTCATAAACCTTGACTACTACCTCTCC	
302	ATTTCTCTGGTGATTTCCTGTGGAGG	To analyze the deletion of *cdc6*
303	CACTAACCTCTGGATTTTCCCGC	
487	GCGGCATGAGGTGTTCACAATGGCGAGAAAGAAAAAGGTTGAGACTGTTGACG	To construct plasmid to replace p*radA* by p*TERMP_00015*
488	CGTCAACAGTCTCAACCTTTTTCTTTCTCGCCATTGTGAACACCTCATGCCGC	
489	GGAGTTTCATTTCCACTGGAAATTTAGCTGAACATCGAAAACCCCAGTAGATTGC	
490	GCAATCTACTGGGGTTTTCGATGTTCAGCTAAATTTCCAGTGGAAATGAAACTCC	
491	TCAG**GGTACC**AGGAAATCACCAGAGAAATATTAGCATTTGTCC	
492	GCTA**AGATCT**GGGGTGAATCAATGAGCCTTGC	
539	TGCTGTCTCTCTGCTAAAGCTCCC	To perform qPCR
j540	TGCTGAAAATAGGGGCTTGGATCC	

The promoter of *RadA* was exchanged with that of *TERMP_00015* using pRD423. This plasmid was constructed using the fusion of three DNA fragments obtained with primer pairs 487/492, 488/489, and 490/491 and was fused using 491/492. The fused DNA fragments were digested by *Bgl*II/*Kpn*I and inserted into pUPH. Details of primers are given in Table 2.

### Transformation methods and strains verification

The transformation of *T. barophilus* was performed as already described in Thiel et al. (24) using 0.2 to 2 µg of plasmid. Verification of the deletion was performed using 7/8 to ensure that non-replicative plasmid used to construct mutant did not stay in the cell, and for *oriC*, *cdc6* mutants, primer pairs outside the construction, 257/258 and 302/303 were used, respectively.

### Marker frequency analysis

DNA was extracted from cultures of *Thermococcus* species at exponential or stationary phase growth (around 3–5 × 10^7^ and 2–3 × 10^8^ cells/mL) or at different points during the growth curves for *T. barophilus* using protocols described previously (24). Library preparation and Illumina sequencing were performed with Novogene, UK. Read mapping was performed with Bowtie2 (35). Normalized average number of reads per position was used to estimate relative replication initiation activity. High read counts were statistically treaded as peaks, and the analysis of the peak area was performed using Origin 2016 (OriginLab Corporation, Northampton, MA, USA) with the peak analyzer tool.

### Optimal pressure growth

For optimal pressure experiments, cells were grown into 15-mL sterile glass vials without gas phase, incubated at 40 MPa of hydrostatic pressure (Thermostated HP/HT incubators Top Industrie, France). All the biological replicates (at least three for each strain) were incubated in the same stainless steel pressure vessels (pressurized by pumping water into them). For marker frequency analysis (pooling several vials from the same reactor was necessary to have enough DNA), cells were harvested by centrifugation (8,000 × *g*, 10 min, 4°C) in mid-exponential (approximately 3–5 × 10^7^ cells/mL) and stationary phases (around 2–3 × 10^8^ cells/mL).

### Determination of cell number for growth kinetics

Cell counts were performed on a Thoma chamber (Preciss, France; surface area: 0.0025 mm^2^, depth: 0.100 mm) and by phase contrast microscopy (Olympus BX60) to verify the cell density during growth kinetics.

### Western blotting on cellular extract

Cells were cultivated as described previously in 30 mL. Cells from strains in the stationary or exponential growth phase were harvested and resuspended in PBS buffer complemented with protease inhibitors (Roche, #05056489001). The cells were disrupted after 5 min in ultrasonic bath, and cellular extracts were collected from the supernatant after centrifugation at 10,000 × *g* for 60 min. The concentrations of total protein were measured by Bradford protein assay (Bio-Rad, #500-0205).

Cell extracts were separated by denaturing electrophoresis (Bio-Rad, #4568094) and transferred onto a PVDF membrane (Bio-Rad, #1704156) during 3 min at 25 V with the Trans-blot turbo transfer system (Bio-Rad, #1704150). The blots were blocked with 5% milk in PBS-T for 60 min and incubated with primary antibody 1:5,000 for 60 min and secondary antibody 1:10,000 for 60 min. Anti-RadA antibodies (gift from Ishino’s lab) were prepared by immunizing rabbits with the recombinant *P. furiosus* RadA. Anti-rabbit IgG HRP (GE Healthcare, NA934V) was used as the secondary antibody. Proteins were visualized with an enhanced chemiluminescence detection system (Thermo Fisher, 34076) and a Chemidoc-XRS image analyzer (Bio-Rad).

### Quantitation

Before transfer, total proteins loaded into the gel were detected by fluorescence with a stain-free imaging system (Bio-Rad). Intensity of protein bands was quantitated by Image Quant software and compared to a reference (WT) (36) to normalize data to total protein in each condition: normalization factor = total WT protein/total P15 protein. The stain-free blot was also analyzed to confirm the transfer quality for each condition. Then, the proteins visualized by chemiluminescence were quantitated to obtain the volume (intensity), and this volume was normalized as follows: normalization volume = volume × normalization factor. After normalization, the percentage of RadA protein was the result of the indicated volume/referenced volume (WT).

### Chromosome number determination by quantitative real-time PCR

Quantitative real-time PCR was performed from the different culture samples taken during the growth in triplicates of the wild strain *T. barophilus* and the mutated strain Δ*oriC* from which the DNA was extracted. A new primer set specific to *RadA* gene was designed: 539 and 540. Primer concentration was optimized to minimize the secondary structure formations and to maximize the reaction efficiency. qPCR reactions were performed in a final volume of 25 µL using PerfeCTa SYBR Green SuperMix ROX (Quanta Bioscience) on a CFX96 Touch Real-Time PCR System (Bio-Rad), 1 ng of DNA template, and 800 nM primers. Forty cycles were performed including one hot-start polymerase activation cycle (95°C, 10 min) and 40 cycles of denaturation (95°C, 15 s) followed by a coupled hybridization and elongation step (60°C, 1 min). Standard curve was obtained from 10-fold serial dilutions (1,000 to 109 copies) of plasmid containing RadA gene from *T. barophilus*. Each reaction was run in triplicates. The quality of qPCR runs was assessed based on melting curves and measured efficiencies; the R of standard curves generated by qPCR and efficiency of the reaction were around 0.999% and 90%, respectively. The qPCR results were expressed in number of chromosomes per cells.

## Data Availability

Data were submitted to NCBI under BioProject accession number PRJNA1047772.

## SUPPLEMENTAL MATERIAL

Fig. S1 mbio.03200-23-s0001.pdf MFA for reference strain during growth.

Fig. S2 mbio.03200-23-s0002.pdf MFA for DeltaOri strain during growth.

Fig. S3 mbio.03200-23-s0003.pdf MFA for RadA^KD^ strain during growth.

Fig. S4 mbio.03200-23-s0004.tif WB 40MPa vs atmospheric pressure.

Legends mbio.03200-23-s0005.docx Legends for supplemental figures.

ASM does not own the copyrights to Supplemental Material that may be linked to, or accessed through, an article. The authors have granted ASM a non-exclusive, world-wide license to publish the Supplemental Material files. Please contact the corresponding author directly for reuse.
