# Supplementary material for: Cooperation between two modes for DNA replication initiation in the archaeon Thermococcus barophilus
Source: mBio. 2024 Feb 29;15(4):e03200-23. doi: 10.1128/mbio.03200-23 (PMC11005403; doi:10.1128/mbio.03200-23)
Supplement: Fig. S1 — MFA for reference strain during growth. [file mbio.03200-23-s0001.pdf]

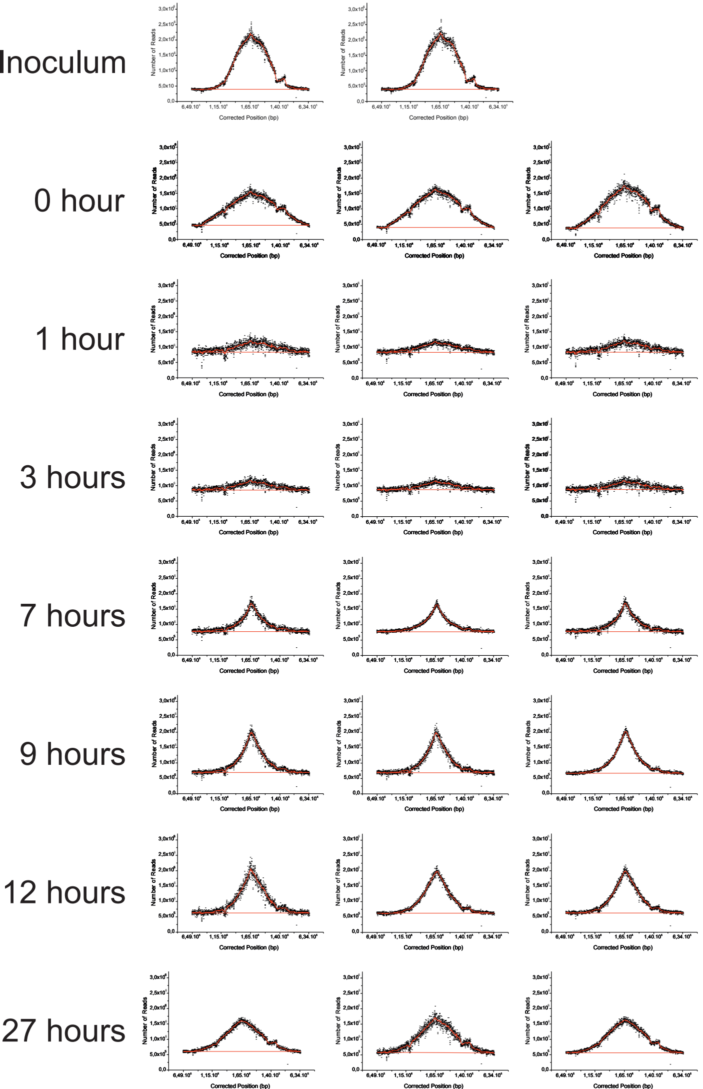
Inoculum
0 hour
1 hour
3 hours
7 hours
9 hours
12 hours
27 hours
Number of Reads
Corrected Position (bp)
3,0x10^6
2,5x10^6
2,0x10^6
1,5x10^6
1,0x10^6
5,0x10^5
0,0
6,49.10^5
1,15.10^6
1,65.10^6
1,40.10^5
6,34.10^5
