# Supplementary figures and images for: Cooperation between two modes for DNA replication initiation in the archaeon Thermococcus barophilus
Source: mBio. 2024 Feb 29;15(4):e03200-23. doi: 10.1128/mbio.03200-23 (PMC11005403; doi:10.1128/mbio.03200-23)

Inoculum

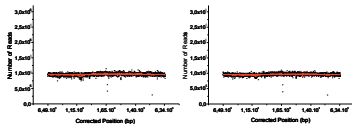

0 hour

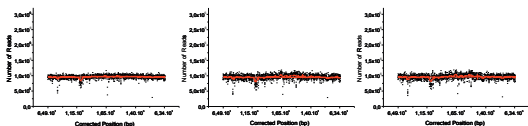

1 hour

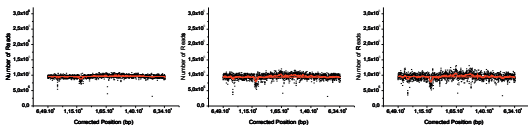

3 hours

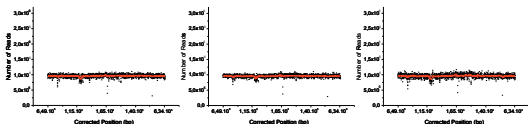

7 hours

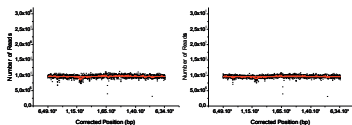

9 hours

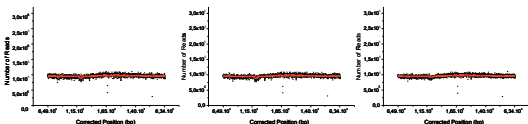

12 hours

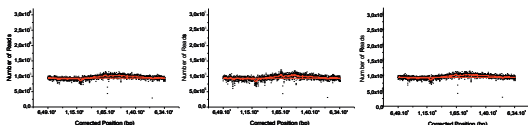

27 hours

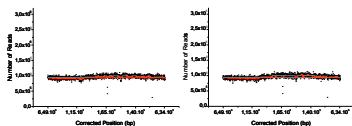

Supplement: Fig. S2 — MFA for DeltaOri strain during growth. [file mbio.03200-23-s0002.pdf]

Inoculum

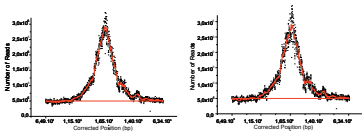

0 hour

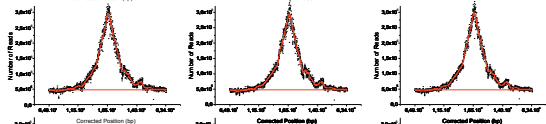

1 hour

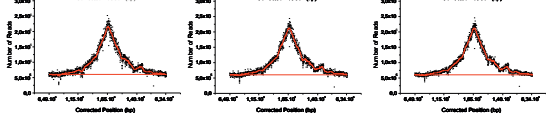

3 hours

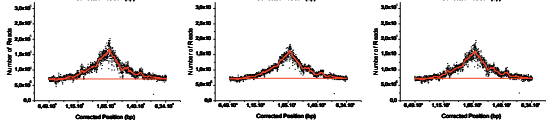

7 hours

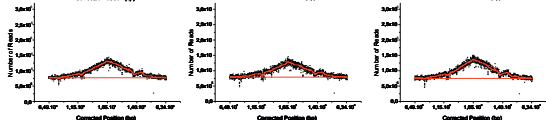

9 hours

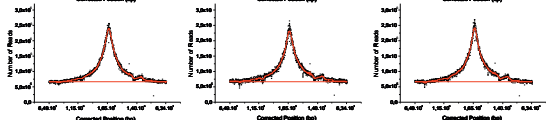

12 hours

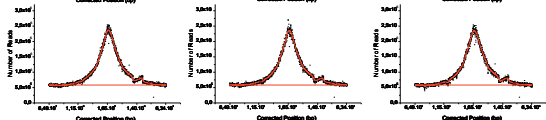

27 hours

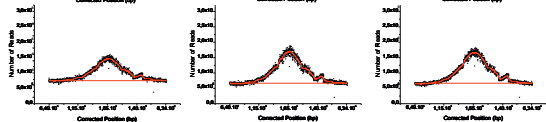

Supplement: Fig. S3 — MFA for RadAKD strain during growth. [file mbio.03200-23-s0003.pdf]

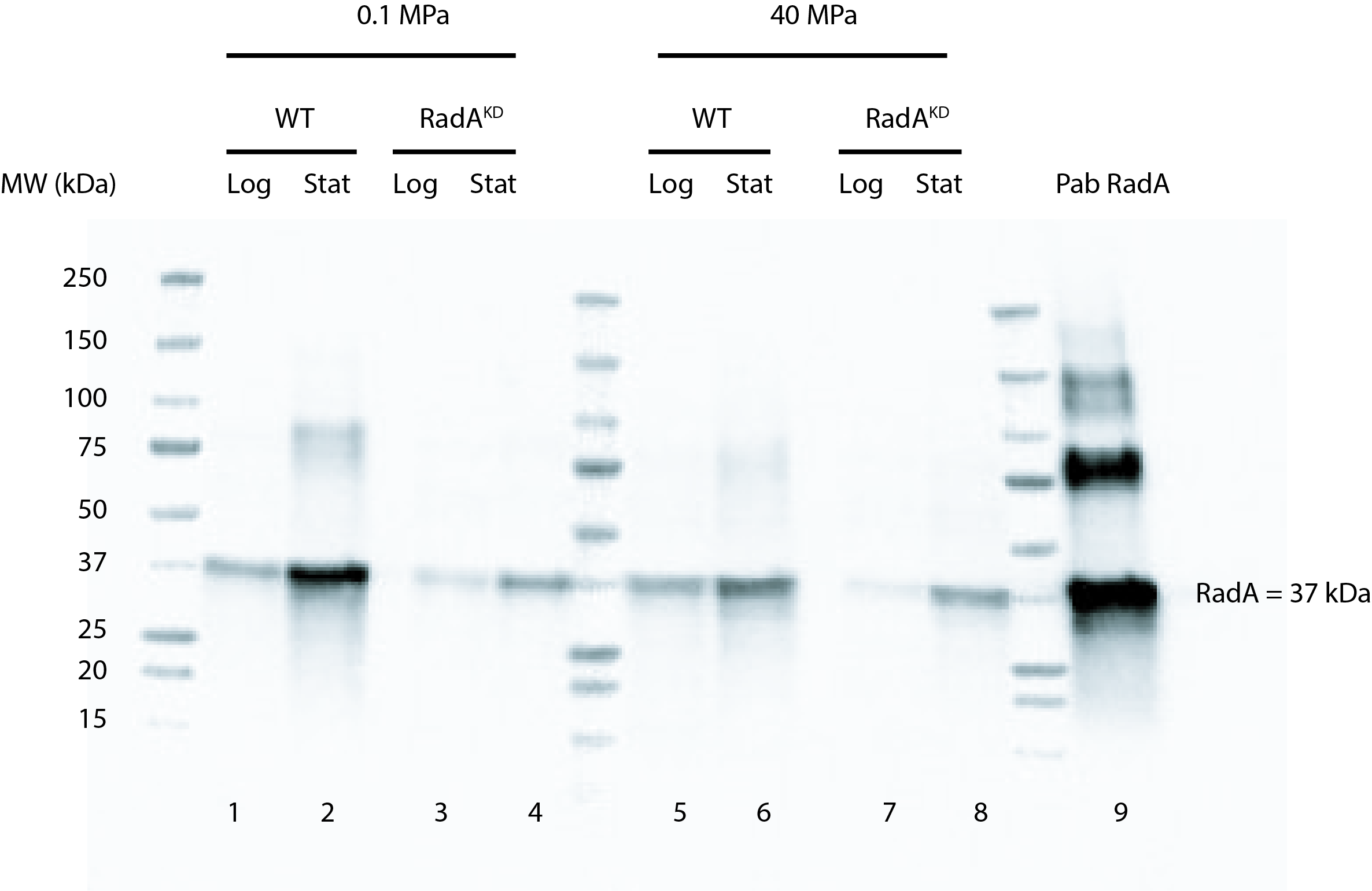

Supplement: Fig. S4 — WB 40MPa vs atmospheric pressure. [file mbio.03200-23-s0004.tif]
