# Supplementary material for: Cooperation between two modes for DNA replication initiation in the archaeon Thermococcus barophilus
Source: mBio. 2024 Feb 29;15(4):e03200-23. doi: 10.1128/mbio.03200-23 (PMC11005403; doi:10.1128/mbio.03200-23)
Supplement: Legends — for supplemental figures. [file mbio.03200-23-s0005.docx]

Supplementary Figure 1: Marker Frequency Analysis at different times of the growth of WT. The red lines represent the one-dimensional Gaussian filter. The horizontal red line was used to calculated the area of the *oriC* peak.

Supplementary Figure 2: Marker Frequency Analysis at different times of the growth of Δ*oriC*. The red lines represent the one-dimensional Gaussian filter. The horizontal red line was used to calculated the area of the *oriC* peak.

Supplementary Figure 3: Marker Frequency Analysis at different times of the growth of RadA^KD^. The red lines represent the one-dimensional Gaussian filter. The horizontal red line was used to calculated the area of the *oriC* peak.

Supplementary Figure 4: Expression of RadA in WT and RadA^KD^ strains at atmospheric pressure and 40MPa. Western blot on RadA in WT and RadA^KD^ strains during exponential and stationary phases at both pressures. Each line was performed using 5 µg of total proteins and a purified RadA from *P. abyssi* was added as control. Here this western was used to compare easily WT and RadA^KD^ strains at both pressure.
